# Switching charge states in quasi-2D molecular conductors

**DOI:** 10.1093/pnasnexus/pgac089

**Published:** 2022-06-13

**Authors:** Yulong Huang, Travis Mitchell, Yixiong Zheng, Yong Hu, Jason B Benedict, Jung-Hun Seo, Shenqiang Ren

**Affiliations:** Department of Mechanical and Aerospace Engineering, University at Buffalo, The State University of New York, Buffalo, NY 14260, USA; Department of Chemistry, University at Buffalo, The State University of New York, Buffalo, NY 14260, USA; Department of Materials Design and Innovation, University at Buffalo, The State University of New York, Buffalo, NY 14260, USA; Department of Mechanical and Aerospace Engineering, University at Buffalo, The State University of New York, Buffalo, NY 14260, USA; Department of Chemistry, University at Buffalo, The State University of New York, Buffalo, NY 14260, USA; Department of Materials Design and Innovation, University at Buffalo, The State University of New York, Buffalo, NY 14260, USA; Department of Mechanical and Aerospace Engineering, University at Buffalo, The State University of New York, Buffalo, NY 14260, USA; Department of Chemistry, University at Buffalo, The State University of New York, Buffalo, NY 14260, USA; Research and Education in energy, Environment and Water (RENEW) Institute, University at Buffalo, The State University of New York, Buffalo, NY 14260, USA

**Keywords:** molecular conductors, charge states, 2D, phase control, stimuli-responsive

## Abstract

2D molecular entities build next-generation electronic devices, where abundant elements of organic molecules are attractive due to the modern synthetic and stimuli control through chemical, conformational, and electronic modifications in electronics. Despite its promising potential, the insufficient control over charge states and electronic stabilities must be overcome in molecular electronic devices. Here, we show the reversible switching of modulated charge states in an exfoliatable 2D-layered molecular conductor based on bis(ethylenedithio)tetrathiafulvalene molecular dimers. The multiple stimuli application of cooling rate, current, voltage, and laser irradiation in a concurrent manner facilitates the controllable manipulation of charge crystal, glass, liquid, and metal phases. The four orders of magnitude switching of electric resistance are triggered by stimuli-responsive charge distribution among molecular dimers. The tunable charge transport in 2D molecular conductors reveals the kinetic process of charge configurations under stimuli, promising to add electric functions in molecular circuitry.

Significance Statement Quasi 2D molecular conductors are intensively investigated for the potential applications of flexible electronic and photonic devices. The existence of multiple metastable phases in quasi-2D molecular conductors feasibly allows a controllable manipulation on matter states. However, the precise control on charge states is exceedingly difficult and hardly studied in a comprehensive method, resulting in a limitation on its further development toward applications. The charge states switching presented in this work demonstrates a successful control on multiple electronic states using several stimuli in a concurrent manner. The phase diagrams of electronic states revealed by cooling rate, current, voltage, and laser irradiation would facilitate a precise control on matter states and help advance device applications based on molecular conductors.

## Introduction

Tuning charge states in molecular nanostructures is an important step to develop molecular electronic circuitry and gain insights into their electrical functions (1). The conformation and dimensionality effects on transport properties of molecular nanostructures are ubiquitous in molecular electronic systems, providing the pathway toward charge state control by different external stimuli (2–7). Electrical charge conductance in molecular entities is a key enabling basis underlying the function of molecular electronics (8, 9); however, it is increasingly clear that controlling the electronic coupling and conductance of molecular electronics is complex as determined by quantum electronic nature (10), while in particular its metallic transport, the hallmark of a decreased electrical resistivity behavior versus temperature, is not typical in disordered molecular systems due to the short mean free path and the localization of charge carriers (11).

The ability to trigger molecular metallicity provides powerful design guidelines to construct molecular circuits to control charge transport (12). Current-induced insulator-metal transition was revealed in potassium 7,7,8,8-tetracyanoquinodimethanane with the formation of a metallic path, indicating a critical role of charge distribution on phase control (13). Molecular crystals based on bis(ethylenedithio)tetrathiafulvalene (abbreviated as ET hereafter) include insulators (14–16), conductors (17–19), and superconductors (20, 21), provide abundant candidates of tunable charge states for phase manipulation (5). Metallicity has been exhibited in our previous work by current control on a 1D molecular ferromagnetic semiconductor (ET)Cu[N(CN)_2_]_2_ (7), where charge interaction affects on phase emergence. The charge crystallization and vitrification in ET dimers are extensively influenced by cooling rate, which dynamically determines the presence of charge crystal, glass, and liquid in *θ*_m_-(ET)_2_TlZn(SCN)_4_ and *θ*-(ET)_2_RbZn(SCN)_4_ (22, 23). Therefore, it is of utmost importance to create molecular building blocks to tune its electronic functionalities and provide quantitative means to establish charge state diagram for potential application of molecular synthetic devices (24). We, thus select quasi-2D layered and exfoliatable electronic crystal *κ*-(ET)_2_Cu[N(CN)_2_](Cl_0.89_Br_0.11_) (abbreviated as *κ*-Cl_0.89_Br_0.11_, see Notes S1 and S2, Supplementary Material) as a prototypical molecular electronic material example (23). The quasi-2D *κ*-Cl_0.89_Br_0.11_ crystal of stimuli-controlled molecular charge states is demonstrated, where its electrical conductance can be tuned toward metallic transport by applying cooling rate, electric current/voltage, and pulsed laser irradiation in a concurrent manner. The phase diagrams of charge states revealed by multiple stimuli provide an accessibly control on charge redistribution in molecular crystals of metastable phases for flexible organic electronics applications (22, 23, 25).

## Results and Discussion

### Exfoliatable layered molecular electronic crystals

**Fig. 1.**
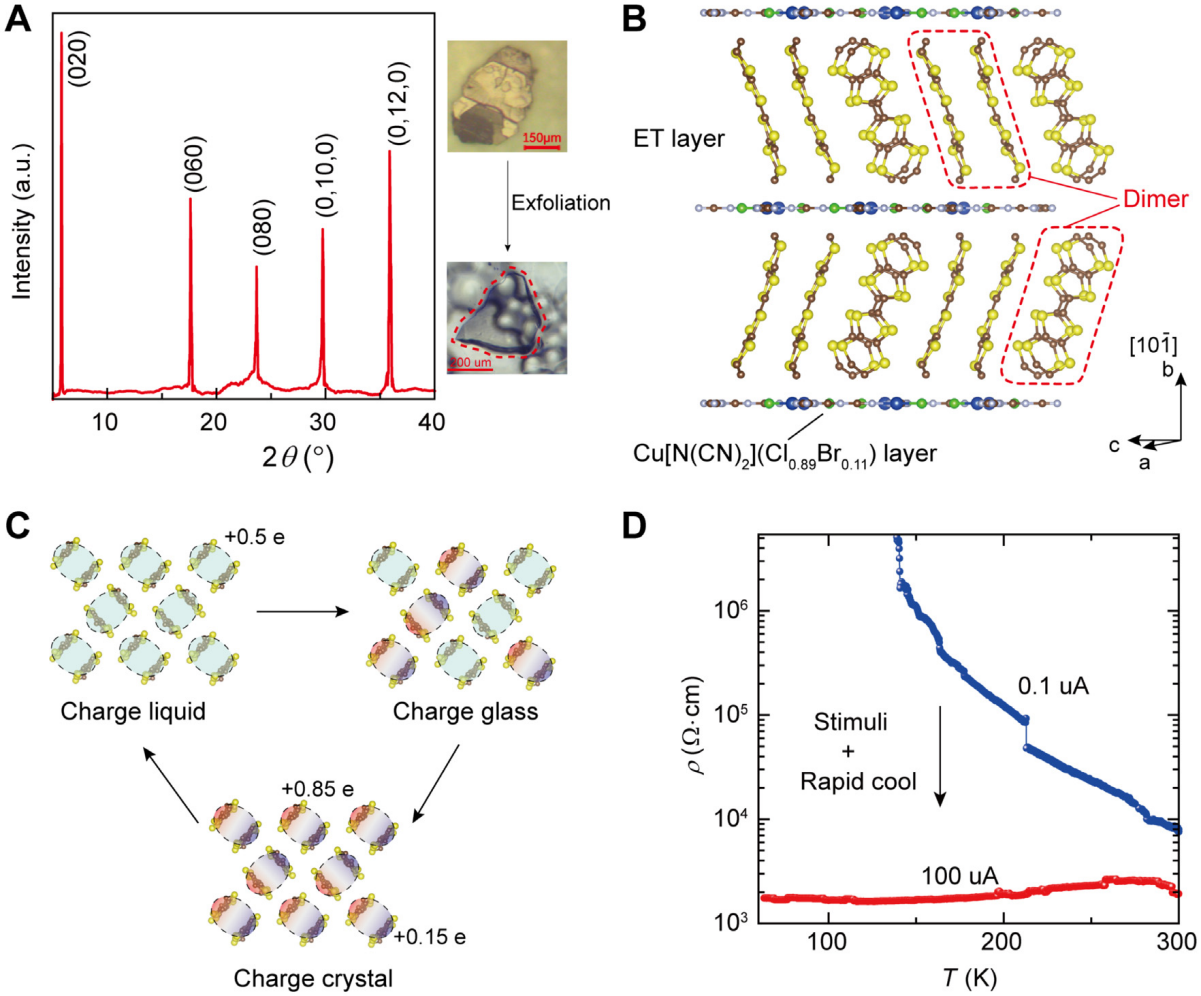
Crystal structure, charge pattern and stimuli effects on 2D *κ*-Cl_0.89_Br_0.11_ crystals for low-temperature metallicity. (A) XRD pattern of *κ*-Cl_0.89_Br_0.11_, typical for ET-based molecular crystals, shows a series of (0*k*0) diffraction peaks (*k* is even), which is the preferred crystallographic orientation along *b*-axis. (040) peak is too weak to observe. The inset is an optical image of a 2D *κ*-Cl_0.89_Br_0.11_ crystal. (B) Crystal structure of *κ*-Cl_1-_*_x_*Br*_x_* system is consisting of conducting ET layer and nonmagnetic insulating Cu[N(CN)_2_](Cl_0.89_Br_0.11_) layer that are alternatively stacking along *b*-axis. (C) Charge redistribution within ET dimers transforms charge states among charge liquid, charge glass, and charge crystal. (D) Low-temperature metallicity is realized by applying external stimuli (here is 100 uA high current) and a rapid sweeping rate.

The electrochemically grown quasi-2D *κ*-Cl_0.89_Br_0.11_ crystals show the preferred 2D growth along (0*k*0) plane (26) revealed by the powder X-ray diffraction (PXRD) pattern and can be mechanically exfoliated into transparent membranes (Figure 1A; Figure S1, Supplementary Material). Single crystal X-ray diffraction (SCXRD) analysis of *κ*-Cl_0.89_Br_0.11_ reveal their lattice consists of two distinct charged layers that alternate along the crystallographic *b*-axis. The conducting layer comprises cationic *κ*-type ET radical dimers, while the nonmagnetic insulating layer comprises Br-doped Cu[N(CN)_2_]Cl^–^ anionic polymer chains (Figure 1B; Figures S2 and S3, Supplementary Material). Structural refinement parameters and details, which include the treatment of the mixed-halide system are presented in Table S1 and Note S2 (Supplementary Material). The as-grown *κ*-Cl_0.89_Br_0.11_ crystal shows ferromagnetic behavior below 15 K (Figure S4, Supplementary Material) which is lower than spin order temperature in *κ*-Cl (27).

### Metallicity in the vicinity of charge crystallization

In *κ*-Cl_0.89_Br_0.11_ crystals, the ET dimer layer donates electron carriers for charge transport, while the Cu[N(CN)_2_](Cl_0.89_Br_0.11_) layer is insulating. The stacking structure of ET molecules and charge distribution within ET dimmers have shown to dominate the dynamic switching of electronic states from stimuli-responsive electron interactions (5, 6, 28, 29, 30). Such control is resulted from the unequal charge redistribution in the ET dimer layer into the ordered or disordered patterns that controls charge crystallization from charge liquid, glass to crystal states (Figure 1C) (22, 25, 31). The *κ*-Cl_0.89_Br_0.11_ crystal behaves as a typical semiconductor with a half-filled band, resulting in an increased resistivity as temperature decreases (Figure 1D). Due to the doping of Br element, *κ*-Cl_0.89_Br_0.11_ crystal is chemically tuned away from Mott insulator *κ*-Cl (32, 33) but a little close to ambient-pressure superconductor *κ*-Br (34), facilitating stimuli control on charge states for metallicity. As decreasing the temperature, the ET dimer formed by face-to-face adjacent molecules dominates low-temperature conducting behavior as a result from charge order degree in the ET dimers (25). By applying a high current of 100 uA under a rapid cooling rate of 3 K/min, we demonstrate a metallic transport behavior in *κ*-Cl_0.89_Br_0.11_ with a monotonic decrease in electrical resistivity versus temperature. The injected charge carriers by high electric current coupled with a rapid cooling, where the nonequilibrium charge crystallization is rapidly quenched, transform *κ*-Cl_0.89_Br_0.11_ into a metallic state at low temperatures. The lowest resistivity of 1.6 kΩ/cm is comparable to the value (0.1 to 10 kΩ/cm) of a flexible field-effect device, where metallicity is induced between 40 and 20 K by the strain effect (33).

### Electric current effect triggering metallicity

**Fig. 2.**
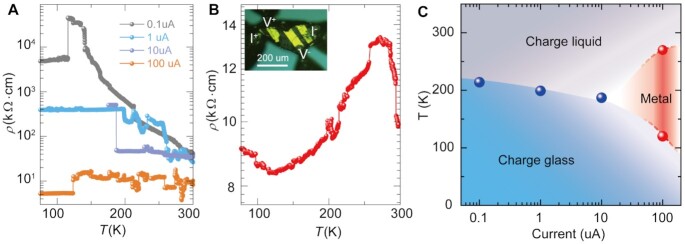
The induced low-temperature metallicity and phase diagram in *κ*-Cl_0.89_Br_0.11_ crystals. (A) A series of electric current (0.1, 1, 10, and 100 uA) are applied to measure the temperature dependent resistivity by cooling that decreases as current increased. The resistivity is almost constant when current is 100 uA. (B) The metallic behavior in low-temperature resistivity is induced between 120 and 270 K by heating up when current is 100 uA. The inset shows the gold-patterned *κ*-Cl_0.89_Br_0.11_ crystal with an electric gap of 32.5 um, a width of 128.8 um, and thickness is 64 um. (C) Temperature-current phase diagram of *κ*-Cl_0.89_Br_0.11_ crystals shows conducting states upon a cooling rate of 3 K/min. The blue dots present the transition temperatures from charge liquid to charge glass at different electric current 0.1, 1, and 10 uA. The red dots present the temperature boundary of metallic state at 100 uA. The gray, green, and red colors represent the different phases of charge liquid, charge glass, and metal, respectively.

The electric current injects charge carriers in *κ*-Cl_0.89_Br_0.11_ to tune its electrical resistivity to access the controlled electronic states. Figure 2(A) shows the temperature dependent resistivity among the range of 10 (4) to 1 kΩ/cm under applied current from 0.1 to 100 uA. The resistivity remains constant with temperature when a current of 100 uA is applied onto *κ*-Cl_0.89_Br_0.11_, while a metallic state with resistivity of 1.6 kΩ/cm emerges at 120 K at a sweeping rate of 3 K/min (Figure 2B). The inset in Figure 2(B) is a typical crystal device with patterned gold electrodes (Figure S5, Supplementary Material). The nonmonotonic temperature dependent resistivity indicates the competition among electronic phases, where a fast-cooling rate induces a quenched nonequilibrium state. Raman spectra indicates that charge evolution of ET molecules develops on charge-sensitive vibration mode under the stimulus of electric current (Figure S6, Supplementary Material). The resistivity decrease (*ρ*_270K_-*ρ*_120 K_)/*ρ*_270 K_ of metallic phase is about 37%, indicating the partial or filamentary metallic phase. The injected charge carriers, together with a rapid sweeping rate to sustain high charge mobility, contribute to the metallic behavior in *κ*-Cl_0.89_Br_0.11_. A current dependent phase diagram is shown in Figure 2(C), indicating the current induced phase transition between charge liquid and glass states. It should be noted that the re-emergence of a semiconducting state could be related to the disordering of ET molecules at lower temperatures. Therefore, to achieve electronic switching, a detailed understanding of electric charge transport transitions in *κ*-Cl_0.89_Br_0.11_ electronic crystal is indispensable.

### Cooling rate effect controlling charge states

**Fig. 3.**
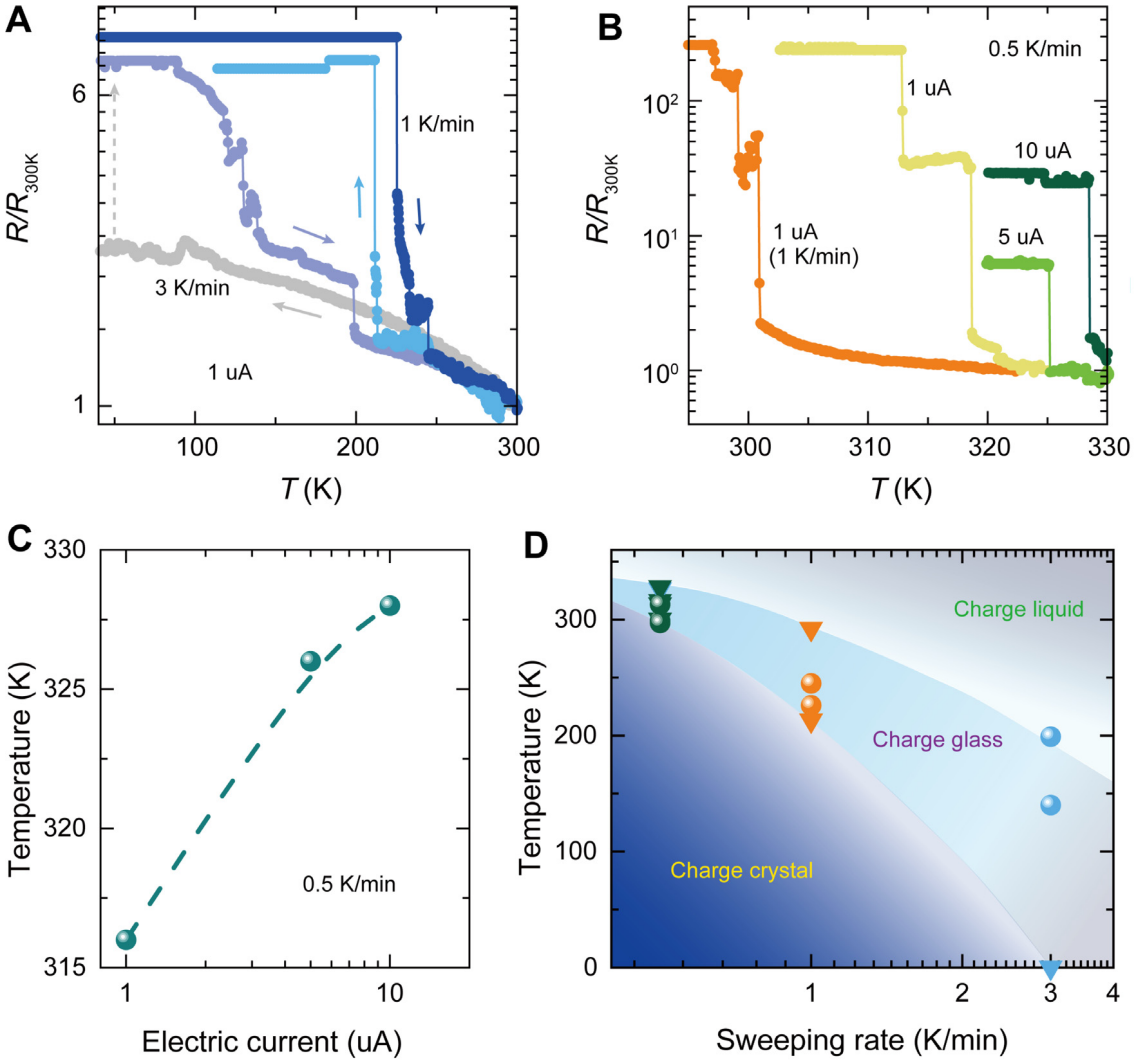
The promoted insulator transition in *κ*-Cl_0.89_Br_0.11_ crystals. (A) At a low electric current, a low-temperature semiconducting behavior is apparent at a cooling rate of 3 K/min. The spontaneous charge crystallization at low temperature turns *κ*-Cl_0.89_Br_0.11_ into an insulating state. The insulator transition temperature is increased by a slower sweeping rate of 1 K/min. (B) The insulator transition can happen at near 300 K on different samples and is enhanced to higher temperature of 318 K by a sweeping rate of 0.5 K/min. The increased electric current of 5 uA and 10 uA allow the insulator transition to occur at 325 K and 328 K, respectively. (C) The current effect on transition temperature at the cooling rate of 0.5 K/min. The dash line is used for eyes guide. (D) Cooling rate versus temperature phase diagram shows charge crystallization.

The cooling rate (6) and electric current play an important role in controlling electronic states of *κ*-Cl_0.89_Br_0.11_. A cooling rate of 3 K/min reveals the spontaneous charge localization of *κ*-Cl_0.89_Br_0.11_ at 40 K (Figure 3A), while at the maintained heating rate of 3 K/min, charge delocalization occurs at 200 K with the decrease of resistance due to thermal activation. However, a slow cooling rate (1 K/min) allows an adequate relaxation to enable charge crystallization at higher temperatures (Figure 3A). The cooling and heating (sweeping) rate effect (6, 35) on charge crystallization is further illustrated in Figure 3(B), where the transition occurs at 300 K and 318 K by 1 K/min and 0.5 K/min, respectively. The transition temperature of *κ*-Cl_0.89_Br_0.11_ can be further changed by increasing electric current (Figure 3B), at which crystals are thermally stable (Figure S7, Supplementary Material). The relationship of transition temperature and electric current at a sweeping rate of 0.5 K/min is plotted in Figure 3(C). The corresponding phase diagram of *κ*-Cl_0.89_Br_0.11_, shown in Figure 3(D), reveals the transition temperature of charge glass and charge crystal as a function of sweeping rate, in which the transition can be diminished beyond the critical sweeping rate 3 K/min. The electronic transitions of *κ*-Cl_0.89_Br_0.11_ induced by charge crystallization are facilitated by a low cooling rate and high electric current.

### Electric voltage and laser modifying charge transport

**Fig. 4.**
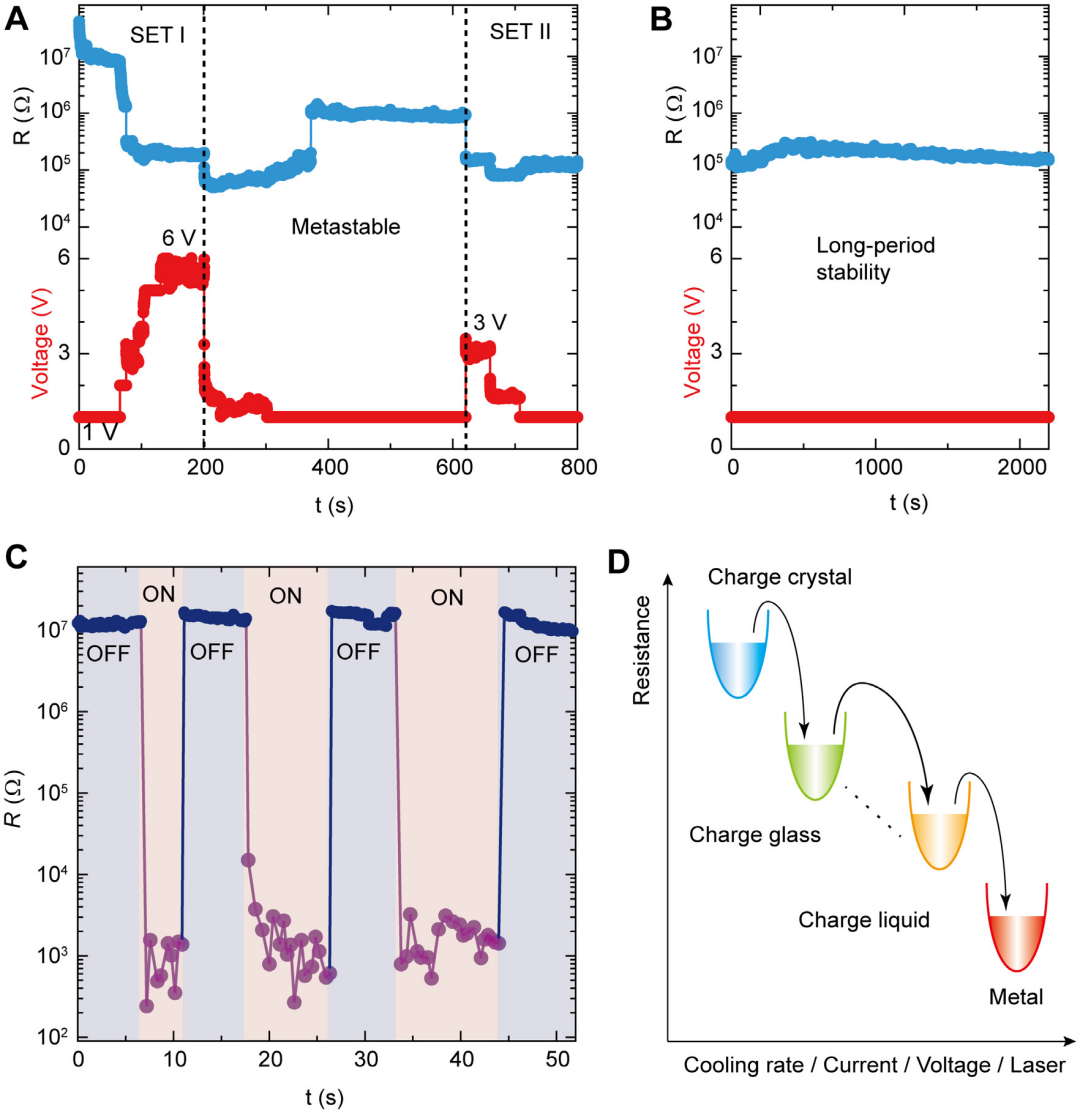
Stimuli effects of voltage and pulsed laser irradiation on *κ*-Cl_0.89_Br_0.11_ crystals. (A) A voltage sequence of 6, 3, and 1.5 V reduces the resistance by two magnitude orders compared to the initial value under 1 V. (B) The low resistance can maintain for a long period of the whole measured time. (C) Pulsed Laser irradiation on/off effect on resistance reveals the reduction of about four magnitude orders. The constant electric current 1 uA was applied. (D) Schematic of charge state switching with external stimuli show the resistance change.

Tunable electronic states can also be accessed at room temperature by applying electric voltage and pulsed laser irradiation. Figure 4(A) illustrates the voltage tuned electrical resistance of *κ*-Cl_0.89_Br_0.11_, with two orders of magnitude decrease (SET I). The metastable resistance under 1 V indicates the low-resistance state is nonequilibrium, which can be maintained with a period of 2,300 s after resetting the voltage (SET II, Figure 4B). The voltage stimulus on *κ*-Cl_0.89_Br_0.11_ provides a high drift force to enhance the mobility of charge carriers. Current versus voltage curves of *κ*-Cl_0.89_Br_0.11_ reveal the transition at a low voltage of 1 V and a linear behavior at high voltages (Figures S8 and S9, Supplementary Material). Figure 4(C) plots the resistance response to a sequence of pulsed laser irradiation (36) at a wavelength of 532 nm, which presents four orders of magnitude decrease of resistance from 10^7^ to 10^3^ Ω. The fast on-off cycles of photons excite *κ*-Cl_0.89_Br_0.11_ under a relatively thin thickness of its surface, enlarging the laser effect on the photon penetration layer. Several cycles of laser shocks confirm the recyclability and stability in resistance values of *κ*-Cl_0.89_Br_0.11_ for both high-resistance and low-resistance states. Due to complex charge pattern, multiple intermediate phases can exist under external stimuli, switching between charge crystal, charge glass, and charge liquid toward metallic transport (Figure 4D). The dynamically controllable electronic states are sensitive to multiple stimuli as presented above, where is accompanied with resistance modification.

### Mechanism of phase control

The key factors for controlling metallicity in *κ*-Cl_0.89_Br_0.11_ are considered as the density of charge carriers and charge mobility. The intrinsic metallicity in molecular electronic materials is rarely observed because of its structural and charge disorders. Here, a rapid sweeping rate allows a limited relaxation of lattice and electronic structure, in which a high charge mobility sustains at low temperatures after quenching (23, 35). Charge crystallization is suppressed at a rapid sweeping rate, while the injected charge carriers facilitate a metallic transport in *κ*-Cl_0.89_Br_0.11_. Thus, a rapid sweeping rate and high electric current synergistically work together to increase charge mobility and density of charge carriers for the presence of metallic transport in molecular electronic crystals. The induced metallic phase competes with the localized state, leading to the resistivity upturn below 120 K. Lattice and charge disorders, as well as intrinsic charge crystallization, contribute to such nonmonotonic behavior of resistivity versus temperature in *κ*-Cl_0.89_Br_0.11_. The multiple charge states of electric transport at above room temperature is also observed in (ET)_2_Br_1.3_I_1.1_Cl_0.6_ organic conductor (17), indicating the doping effect may contribute to multiple metastable phases. Voltage stimulus exhibits a promising ability to control charge states, which also imply *κ*-Cl_0.89_Br_0.11_ is an appropriate material candidate for memory devices (31, 37). Light irradiation can induce current oscillations and melt or freeze charge motion in molecular solids (38, 39), illustrating the dynamic tuning of charge states in *κ*-Cl_0.89_Br_0.11_ and its potential applications on phototransistors (40). Since ferromagnetic order and possible strain-induced diamagnetism were observed in *κ*-Cl_0.89_Br_0.11_ (Figure S10, Supplementary Material), the stimuli effects on low temperature magnetic properties are worth further investigation.

## Conclusion

In summary, we describe the tunable charge transport in *κ*-Cl_0.89_Br_0.11_ electronic crystals by utilizing external stimuli, based on charge mobility and density of charge carriers. Charge phase diagram in *κ*-Cl crystals is established as a function of cooling rate, electric current and voltage, and pulsed laser stimuli to trigger the electronic phase transition and metallic phase. The voltage and pulsed laser irradiation are shown to reduce the resistance of *κ*-Cl_0.89_Br_0.11_ by four orders of magnitude. This work provides a pathway to dynamically control charge states including metallic phase in molecular electronic crystals for potential applications on organic electronic devices.

## Experimental Procedures

### Electrolyte preparation

The preparation process of electrolyte for the growth of *κ*-Cl_0.89_Br_0.11_ single crystals is similar to our previous work on (BEDT-TTF)Cu[N(CN)_2_]_2_ wires (7). First, 15.3 mg CuCl powder (Sigma-Aldrich, 97%) and 60.8 mg PPh_4_N(CN)_2_ with a little PPh_4_Br were dissolved into 15 ml 1,1,2-trichloroethane (Sigma-Aldrich, 97%, TCA) solvent (25 ul H_2_O). After 30 min ultrasonication and 2-hour stirring, 14.4 mg bis(ethylenedithio)tetrathiafulvalene (TCI, BEDT-TTF) was added for 2 more hours of stirring. A clean light-yellow solution was obtained for electrolyte after filtering.

### Growth of κ-Cl_0.89_Br_0.11_ single crystals

As for the synthesis of traditional quasi-2D ET-based molecular crystals, the low constant current (0.1 to 0.2 uA) and Pt electrodes are preferred to guide 2D nucleation and flexibly tune the growth process. Thin quasi-2D *κ*-Cl_0.89_Br_0.11_ crystals can be obtained on the Pt electrode wire when the low constant current is applied for 1 month.

### Structural and morphologic characterizations

X-ray diffraction pattern was obtained on the Rigaku Ultima IV (40 kV, 44 mA, Cu Kα) from 5° to 50°. Field Emission Scanning Electron Microscope (FESEM) Carl Zeiss AURIGA (200 kV) was utilized to get the topological morphology of *κ*-Cl_0.89_Br_0.11_. The element analysis and mapping in *κ*-Cl_0.89_Br_0.11_ were conducted on Oxford Energy-dispersive X-ray Spectrometer (EDS).

### SCXRD methods

Bruker SMART APEX II CCD diffractometer was utilized to detect X-ray diffraction signal of a single crystal mounted on the tip of a glass fiber with oil, which was equipped with a rotating anode source (Mo-Kα radiation, λ = 0.71073 Å). The detector distance from the crystal is 40.00 mm and the 2θ-angle is −25°. At different φ-angles (φ = 0° to 288° in 72° increments), five 180° ω-scans (step ∼ 0.5°) collected a total of 1,800 frames that nominally covered complete reciprocal space for structure refinement. SAINT (version 8.40A) was used for data reduction, and SADABS version 2016 (41) completed a multiscan absorption correction. Space-group determination was conducted by using the XPREP utility in SHELXTL (42). Using Olex2 (43), the structure was solved with ShelXT (44) using intrinsic phasing and refined with ShelXL (45) using least squares minimization (full-matrix least-squares on F^2^).

### Low temperature electrical transport measurements

The low-temperature resistivity of *κ*-Cl_0.89_Br_0.11_ crystal was measured on a Janis low temperature system (CCS-150) with helium gas compressor (CTI-Cryogenics, Helix Technology Corp.). Sample temperature was controlled by Lake Shore 331 cryogenic temperature controller. Data measurement and collection were conducted by Keithley 2450. All data from the instruments were automatically read by the Labview program. The gold electrode pattern of 50 nm thick was deposited onto *κ*-Cl_0.89_Br_0.11_ crystals by e-beam evaporation using a copper grid mask. The deposited pattern for electric current effect has a voltage gap of 32.5 um and a width of 128.8 um; the crystal thickness is 64 um. Silver epoxy was used to contact gold pattern with gold wires for the four-probe measurement. The proper coverage of gold pattern by silver epoxy allows stable connections for measurements.

### Magnetic susceptibility and hysteresis loop measurements

Temperature dependent magnetic susceptibility and magnetic hysteresis loop were measured on Physical Properties Measurement System EverCool II (Quantum Design) equipped with a vibrating-sample magnetometry.

### Spectroscopy measurements

Raman spectroscopy measurements were carried on Renishaw inVia Raman Microscope with an excitation wavelength of 514 nm controlled by a cryogenic programmable intelligent temperature controller (MercuryiTC, Oxford Instruments).

### Pulsed laser irradiation

The Nimma-900 laser system from Beamtech Optronics was used to irradiate crystals under a pulsed laser (wavelength 532 nm, pulse energy 400 mJ, pulse width 7 to 9 ns, repetition rate 10 Hz, beam diameter 9 mm). Constant electric current 1 uA was applieds.

## Supplementary Material

pgac089_Supplemental_File

## Data Availability

All data is included in the manuscript and supporting information.
