# Supplementary material for: Switching charge states in quasi-2D molecular conductors
Source: PNAS Nexus. 2022 Jun 13;1(3):pgac089. doi: 10.1093/pnasnexus/pgac089 (PMC9896912; doi:10.1093/pnasnexus/pgac089)
Supplement: pgac089_Supplemental_File [file pgac089_supplemental_file.pdf]

Supplementary Information

Switching charge states in quasi-2D molecular conductors

Yulong Huang[1], Travis Mitchell[2], Yixiong Zheng[3], Yong Hu[1], Jason B. Benedict[2], Jung-Hun Seo[3], and Shenqiang Ren[1,2,4,*]

[1.] Department of Mechanical and Aerospace Engineering, University at Buffalo, The State University of New York, Buffalo, NY, 14260, USA

[2.] Department of Chemistry, University at Buffalo, The State University of New York, Buffalo, NY, 14260, USA

[3.] Department of Materials Design and Innovation, University at Buffalo, The State University of New York, Buffalo, NY 14260, USA

[4.] Research and Education in energy, Environment and Water (RENEW) Institute, University at Buffalo, The State University of New York, Buffalo, NY, 14260, USA

*E-mail: shenren@buffalo.edu

This PDF file includes:

1. Morphology and element analysis of κ-$Cl_{0.89}Br_{0.11}$ crystals.
2. Crystal structure analysis of κ-$Cl_{0.89}Br_{0.11}$ crystals.
3. Ferromagnetic property, electrode pattern and thermal gravimetric analysis of κ-$Cl_{0.89}Br_{0.11}$ crystals.
4. Current versus voltage curves of κ-$Cl_{0.89}Br_{0.11}$ crystals.
5. Diamagnetic behavior of κ-$Cl_{0.89}Br_{0.11}$ crystals.
Figs. S1 to S10
Table S1
References

Supplementary Note S1. Morphology and element analysis of κ-$Cl_{0.89}Br_{0.11}$ crystals.

The two-dimensional (2D) κ-$Cl_{0.89}Br_{0.11}$ crystals were electrochemically grown on a Pt electrode that usually takes one month. The morphologies of κ-$Cl_{0.89}Br_{0.11}$ crystals are mainly in a shape of rhombus (Figure S1a-S1e), and occasionally present square (Figure S1f). Through the optical images, the crystal boundaries and layered structure are clearly seen in a macroscopic scale since κ-$Cl_{0.89}Br_{0.11}$ grew from multiple nucleation centers. The fresh crystals are likely metallic shiny under optical microscope, indicating the flat surface and abundant charge carriers. The 2D layered κ-$Cl_{0.89}Br_{0.11}$ crystals can be mechanically exfoliated by using Scotch tape or Blue tape, showing very flat mirror-like surfaces (Figure 1g-1i).

Figure S2 presents the element analysis results on a κ-$Cl_{0.89}Br_{0.11}$ crystal by Energy-dispersive X-ray spectrometer. All elements including C, N, S, Cu, Cl and Br were detected and quantitively analyzed for their weight and atomic ratios, where atomic ratio of Cl to Br is 0.89 : 0.11. A structural model (See below Supplementary Note 2) results in an atomic ratio of Cl to Br of 0.85:0.15 that is consistent with the EDS result. Thus, we assign the chemical formula as κ-$(ET)_2Cu[N(CN)_2](Cl_{0.89}Br_{0.11})$, which is abbreviated as κ-$Cl_{0.89}Br_{0.11}$. The element mapping on the κ-$Cl_{0.89}Br_{0.11}$ crystal confirmed the uniformity of all elements throughout the whole crystal, where each element mapping result shows the same characteristic shape in color similar to the morphology of the crystal.

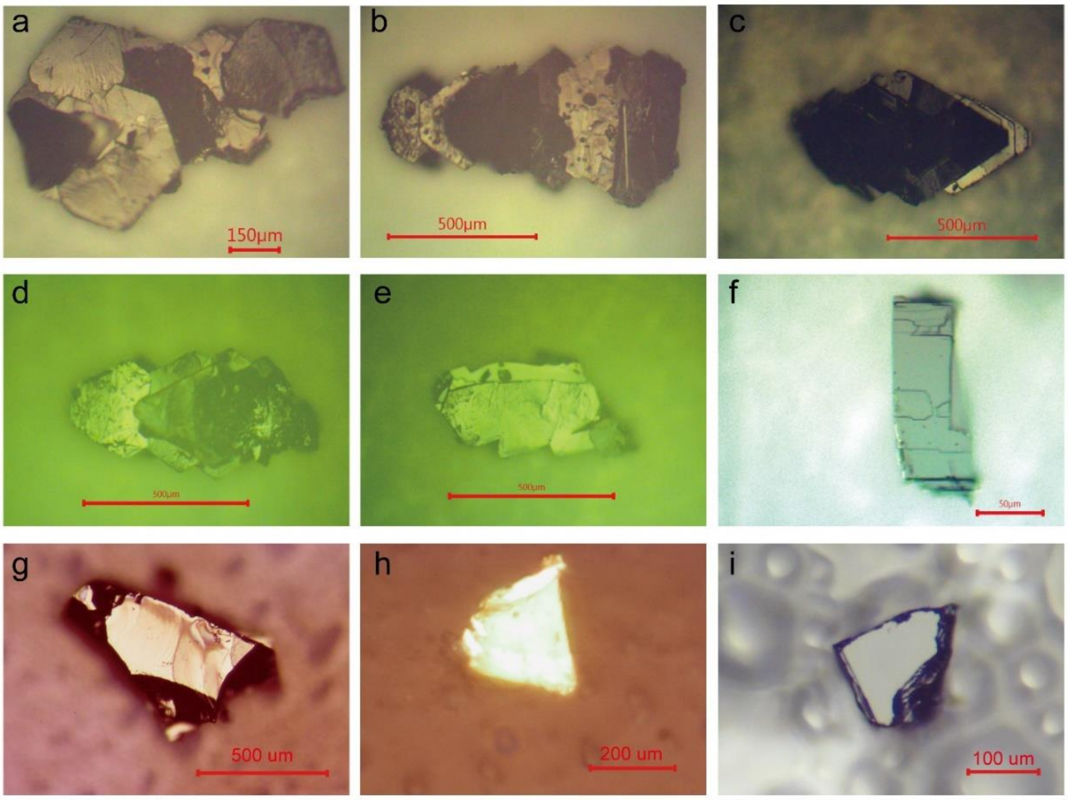

Figure S1. Optical images of κ-$Cl_{0.89}Br_{0.11}$ crystals show the 2D nature. (a-f) as-grown samples. (g-i) cleaved samples.

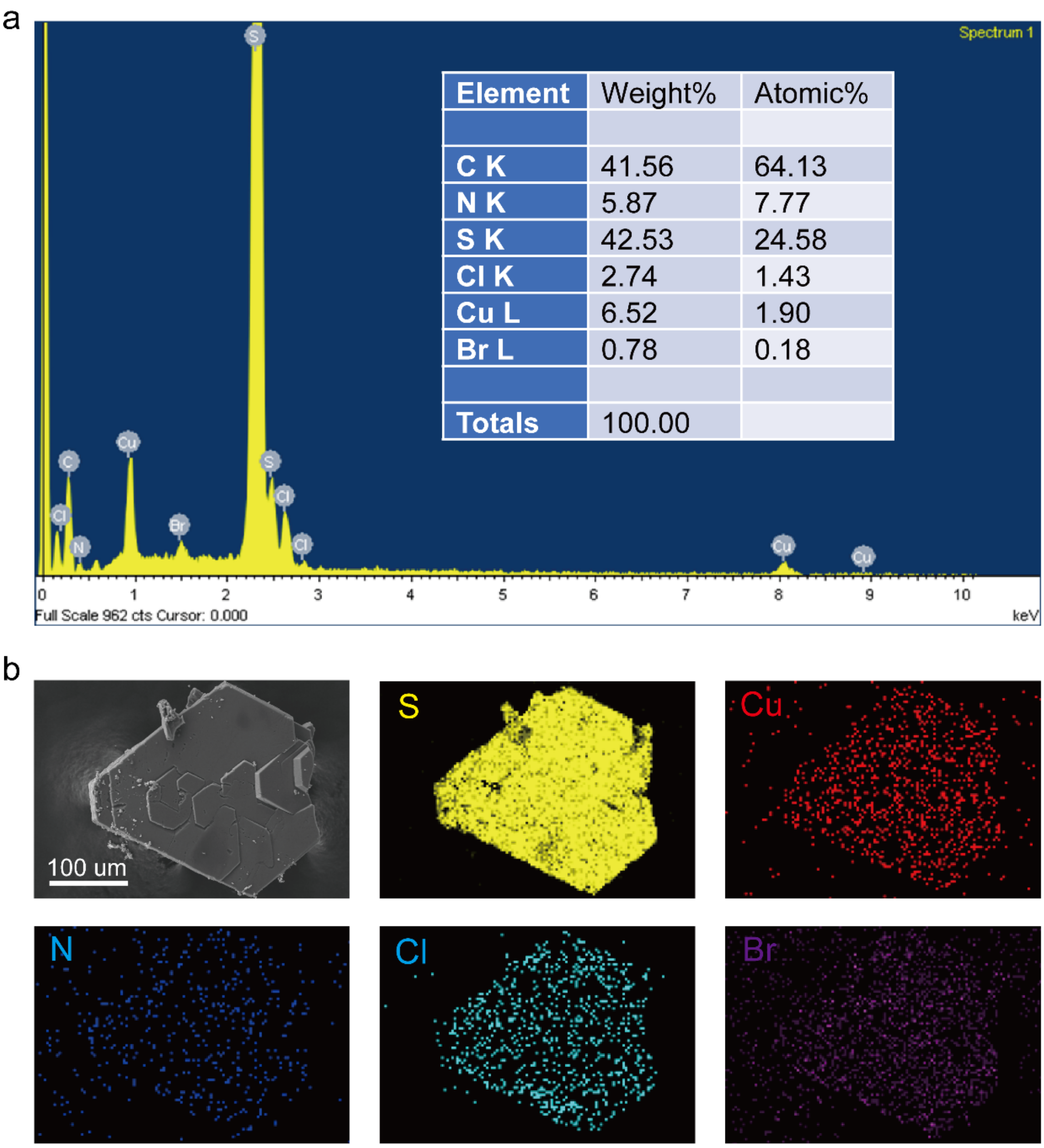

Figure S2. Element analysis and mapping of *κ*-Cl$_{0.89}$Br$_{0.11}$ crystals. (a) Energy-dispersive X-ray Spectra and analysis indicate elements of C, N, S, Cl, Cu, and Br and their ratio. The Cl and Br atomic ratio is calculated as 0.89 : 0.11.

Supplementary Note S2. Crystal structure analysis of κ-$Cl_{0.89}Br_{0.11}$ crystals.

Part 1. Single-crystal structure refinement

One of the ethylene groups in the ET radicals was disordered and modeled appropriately. The mixed halide system for the κ-$Cl_{0.89}Br_{0.11}$ crystals was modeled in three different ways. Originally, the model did not include a bromine atom which yielded an R_1 value of 6.12% with Cu-Cl bond length of 2.214(3) Å. A Q peak along the Cu-Cl bond was then assigned as a bromine atom. The occupancies of the bromine and chlorine atom were refined freely, and the sum of their occupancies was set equal to one. The position of each halide atom was refined independently, but the anisotropic displacement parameters (ADPs) for the two halide atoms were set equal using the SHELX command EADP. This second model yielded an R1 value of 5.85%, Cu-Cl and Cu-Br bond lengths of 2.19(2) Å and 2.30(5) Å respectively, and an atomic ratio of Cl to Br of 0.85:0.15. Finally, the positional parameters for the two halide atoms were set equal using the SHELX command EXYZ which yielded an R1 value of 5.86%, an average Cu-Cl and Cu-Br bond length of 2.22(3) Å, and an atomic ratio of Cl to Br of 0.85:0.15. The latter two models both have an atomic ratio of Cl to Br that is consistent with the atomic ratio calculated from the EDS analysis. Ultimately, the latter model was chosen as the simpler model with the highest data to parameter ratio. The latter model was also consistent with the model for the previously reported κ-$Cl_{0.5}Br_{0.5}$ crystal structure[1]. Supplementary Table 1 lists the crystallographic data of the latter structural model with an atomic ratio of Cl to Br of 0.85:0.15.

Supplementary Table S1. Crystallographic data and structure refinement for *κ*-$Cl_{0.89}Br_{0.11}$ crystals

Identification code	$(ET)_2Cu[N(CN)_2]Cl_{0.85}Br_{0.15}$
Empirical formula	$C_{22}H_{16}Br_{0.15}Cl_{0.85}CuN_3S_{16}$
Formula weight	941.11
Temperature/K	296.15
Crystal system	orthorhombic
Space group	Pnma
a/Å	12.9506(18)
b/Å	29.902(4)
c/Å	8.4713(12)
α/°	90
β/°	90
γ/°	90
Volume/$Å^3$	3280.5(8)
Z	4
$\rho_{calc}g/cm^3$	1.905
μ/mm^{-1}	1.965
F(000)	1895.0
Crystal size/mm^3	0.04 × 0.03 × 0.02
Radiation	MoKα (λ = 0.71073)
2Θ range for data collection/°	2.724 to 49.424
Index ranges	$-14 \leq h \leq 15$, $-25 \leq k \leq 35$, $-9 \leq l \leq 9$
Reflections collected	20210
Independent reflections	2846 [R_{int} = 0.0903, R_{sigma} = 0.0657]
Data/restraints/parameters	2846/0/207
Goodness-of-fit on F^2	1.114
Final R indexes [I>=2σ (I)]	R_1 = 0.0586, wR_2 = 0.1480
Final R indexes [all data]	R_1 = 0.0851, wR_2 = 0.1720
Largest diff. peak/hole / e $Å^{-3}$	0.77/-0.65

Supplementary Part 2. Molecular vibration spectra

Polarized Raman spectra were conducted on exfoliated κ-$Cl_{0.89}Br_{0.11}$ crystals to identify molecular fingerprints of ET vibrational modes. Figure S3 plots the polarized Raman spectra with frequency shift from 100 cm^{-1} to 3200 cm^{-1} along three different directions. The Raman laser excitation of a wavelength of 514 nm is horizontally polarized. Two main Raman active modes from ET molecular vibration v_2 = 1495.0 cm^{-1} and v_3 = 1467.3 cm^{-1} are illustrated in the inserted figure, in which the frequencies do not change with the laser projection direction.

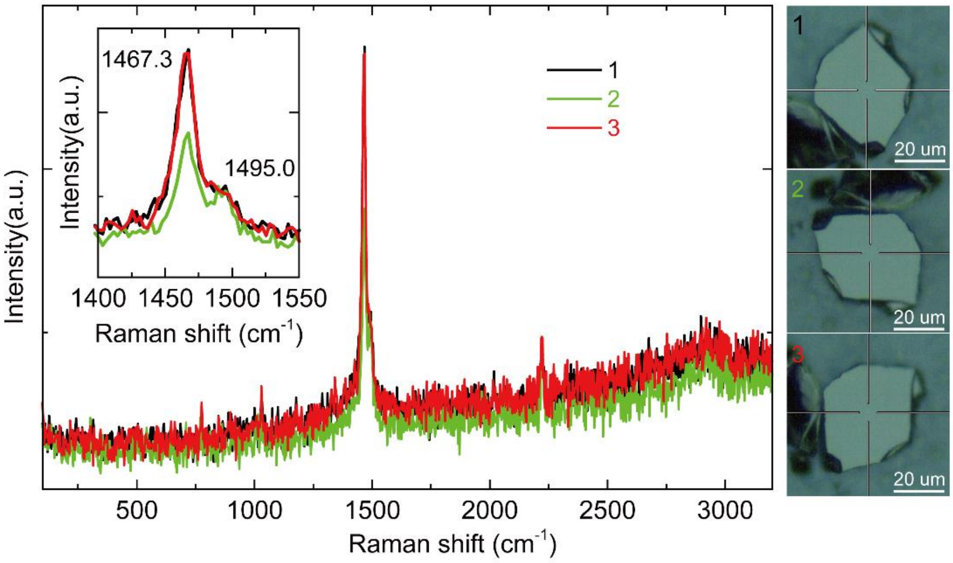

Figure S3. Raman spectra of a cleaved κ-$Cl_{0.89}Br_{0.11}$ crystal with polarized laser projected along three different orientation of the sample.

Supplementary Note S3. Ferromagnetic property, electrode pattern and thermal gravimetric analysis of κ-$Cl_{0.89}Br_{0.11}$ crystals.

Figure S4a shows magnetic properties of pristine κ-$Cl_{0.89}Br_{0.11}$ crystals. At low temperature, a ferromagnetic behavior of upturn in magnetic susceptibility was observed below 15 K that is lower than the order temperature of 23 K in κ-Cl crystals. Magnetic hysteresis loop measured from -1000 Oe to 1000 Oe confirmed ferromagnetic or ferrimagnetic order in κ-$Cl_{0.89}Br_{0.11}$ crystals. Even though the existence of magnetic order points to the similarity between κ-Cl and κ-$Cl_{0.89}Br_{0.11}$ crystals, they also differ by the order temperature that may be attributed to the Br doping effect.

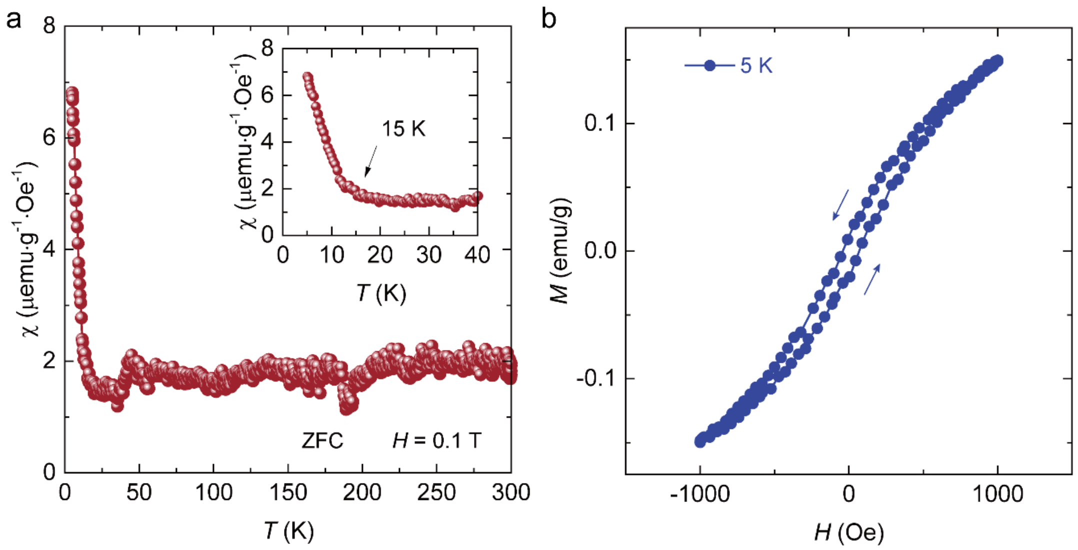

Figure S4. Magnetic properties of κ-$Cl_{0.89}Br_{0.11}$ crystals. (a) Temperature dependent magnetic susceptibility under a magnetic field of 0.1 T indicates susceptibility upturn below 15 K and near constant value at higher temperature. (b) Magnetic hysteresis loop at 5 K confirms the ferromagnetic behavior. The arrow indicates the hysteresis direction.

The electric transport measurements under external stimuli were taken on gold-pattern κ-$Cl_{0.89}Br_{0.11}$ crystals. Figure S5a shows the copper grid mask with hole with of 48 um and bar width of 35 um, which is used to cover crystals during gold evaporation. Gold electrode bars will be deposited in the gaps of copper grid, as shown in Figure S5b. The gold-patterned crystal with four-probe electrodes is presented in Figure S5c, which shows very uniform electrode gap. The gold pattern deposited by e-beam evaporation allows a good contact on $Cl_{0.89}Br_{0.11}$ crystals that is critical for electric transport study under stimuli. Figure S6 indicates Raman spectra with applied current from 0 uA to 100 uA show the charge-sensitive mode v_2 shift from 1498 cm^{-1} to 1492 cm^{-1}.

Figure S7 plots thermal gravimetric analysis of κ-$Cl_{0.89}Br_{0.11}$ crystals, where weight loss occurred at several temperatures 460 K, 515 K, 563 K, and 793 K. Thus, there was no structural damage in κ-$Cl_{0.89}Br_{0.11}$ crystals when electric transport measurement was conducted up to 330 K. As for the tiny kink of derivative of weight at 324 K, the weight was increasing slowly a little that may relate to unbalanced initial state at near room temperature.

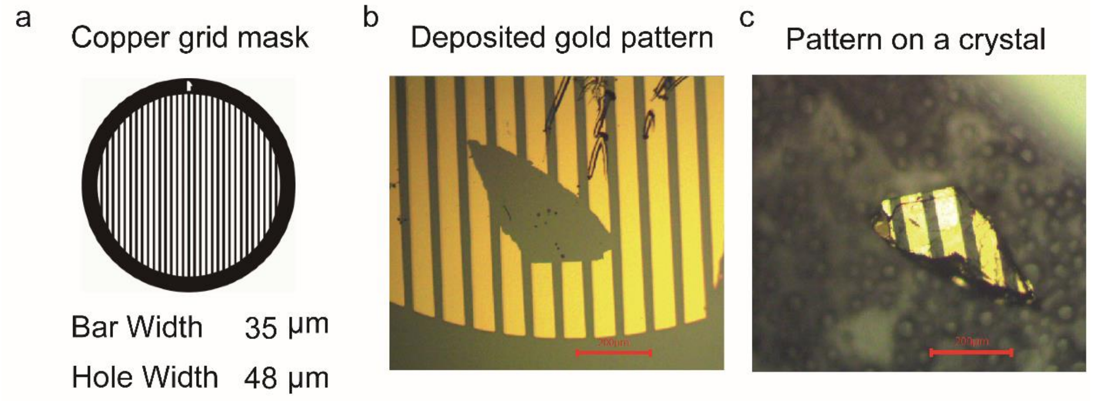

Figure S5. Gold electrode pattern of κ-$Cl_{0.89}Br_{0.11}$ crystals. (a) The copper grid mask with a gap of 48 um is used to cover samples when gold is evaporated. (b) The deposited gold pattern based on the copper grid. (c) The gold pattern of four probes on a crystal.

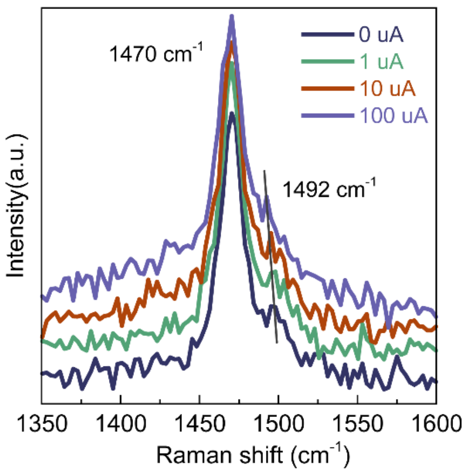

Figure S6. Raman spectra were measured with applied current from 0 uA to 100 uA show the charge-sensitive mode v_2 shift from 1498 cm^{-1} to 1492 cm^{-1}.

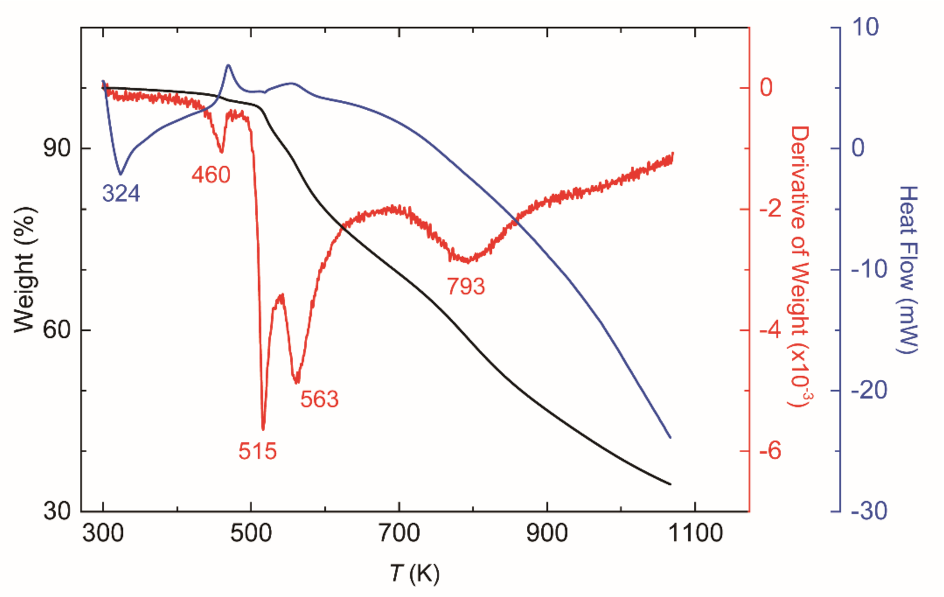

Figure S7. Thermal gravimetric analysis of κ-$Cl_{0.89}Br_{0.11}$ crystals.

Supplementary Note S4. Current versus voltage curves of κ-$Cl_{0.89}Br_{0.11}$ crystals.

Voltage stimulus has shown its tunability on charge conductance states in $Cl_{0.89}Br_{0.11}$ crystals according to the argument in the main text. Here, current versus voltage (*I*-*V*) curves were studied for a further understanding on the evolution of charge state. Figure S8a plots the *I*-*V* curves of positive bias from zero to 20 V at 300 K, where the almost linear relationship is apparent. The inset in Figure S8a shows the current jumps at near 2 V switching from a high resistive state to a low resistive state, which is the feature of resistive memories. The small switching voltage is also indicating the feasible tunability of charge states for metallicity that is also applicable at 280 K (Figure S9). The *I*-*V* curves are linear even sweeping range is extended to 200 V.

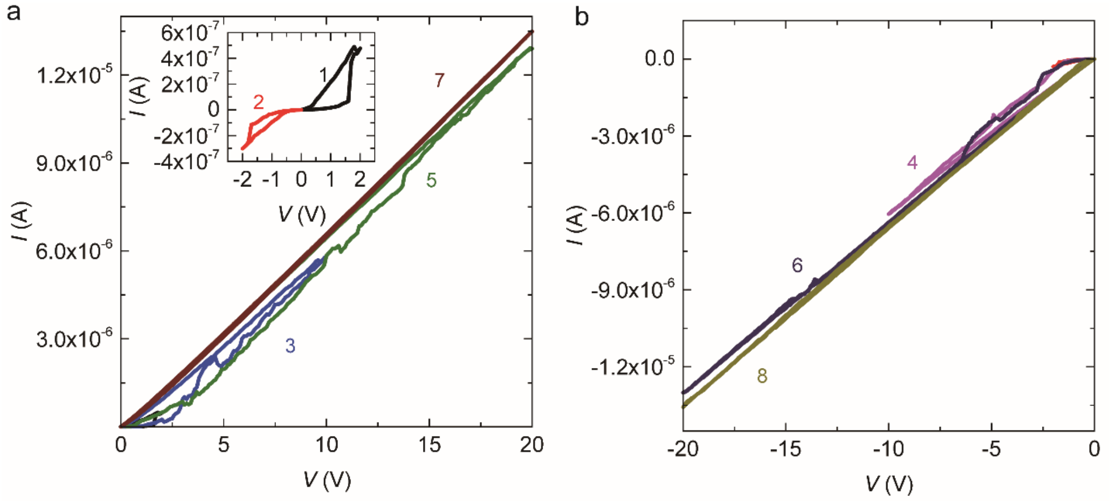

Figure S8. Current versus voltage curves of κ-$Cl_{0.89}Br_{0.11}$ crystals for different voltage ranges at 300 K. The number is the measurement order. The inset shows the IV transition at near 2 V.

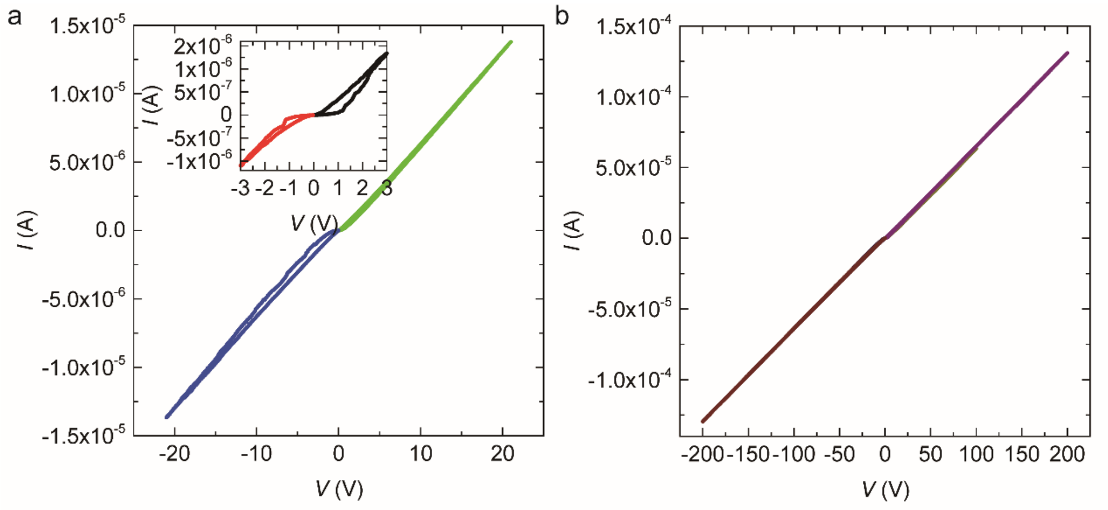

Figure S9 Current versus voltage curves of *κ*-$Cl_{0.89}Br_{0.11}$ crystals for different voltage ranges at 280 K. The number is the measurement order. The inset shows the IV transition at near 1 V.

Supplementary Note S5. Diamagnetic behavior of κ-$Cl_{0.89}Br_{0.11}$ crystals.

In all grown κ-$Cl_{0.89}Br_{0.11}$ crystals, diamagnetic behavior was also observed. At magnetic field of 50 Oe, the diamagnetic transition near 12 K is consistent in zero-filed-cooling and field-cooling curves (Figure S10a). The transition goes lower temperature with increasing magnetic field to 0.01 T and 0.1 T, and then disappears at higher magnetic field (Figure S10b).

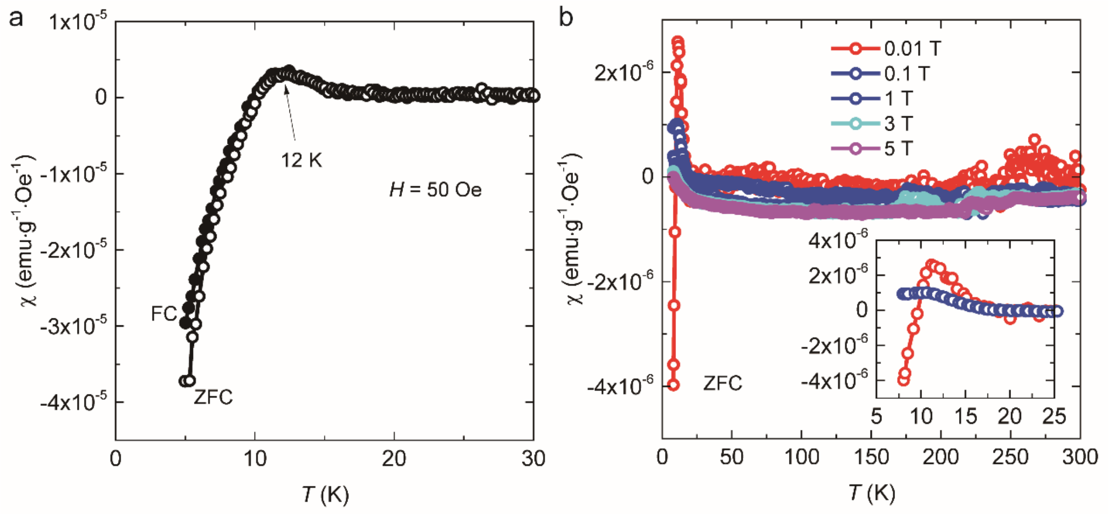

Figure S10 Magnetic properties of κ-$Cl_{0.89}Br_{0.11}$ crystals. (a) Diamagnetic behavior is shown under zero magnetic field cooled and Field cooled susceptibility at 50 Oe. (b) The temperature dependent magnetic susceptibility from 8 K to 300 K are measured under a series of magnetic fields.

Reference

1. Kushch, N. D.; Buravov, L. I.; Khomenko, A. G.; Yagubskii, E. B.; Rosenberg, L. P.; Shibaeva, R. P. *Synthetic Metals* **1993,** 53, (2), 155-160.
